# Epidemiological Characteristics of Dengue Infection in Bangladesh: A Systematic Review

**DOI:** 10.3390/ijerph23020235

**Published:** 2026-02-13

**Authors:** Md Moustafa Kamal, Tsheten Tsheten, Rashidul Haque, Syeda Zakia Hossain

**Affiliations:** 1 School of Health Sciences, Faculty of Medicine and Health, University of Sydney, Sydney, NSW 2006, Australia; zakia.hossain@sydney.edu.au; 2 National Centre for Epidemiology and Population Health, The Australian National University, Acton, Canberra, ACT 0200, Australia; tsheten.tsheten@anu.edu.au; 3 International Centre for Diarrheal Disease Research, Dhaka 1212, Bangladesh; rhaque@icddrb.org

**Keywords:** dengue, infectious disease, serotypes, symptoms, Bangladesh

## Abstract

Background: Dengue infection (DI) is a mosquito-borne arboviral disease primarily transmitted by infected female *Aedes* mosquitoes. In Bangladesh, DI poses a substantial public health challenge with recurrent outbreaks and rising incidence rates. This systematic review assesses the epidemiological characteristics of dengue infection in Bangladesh, focusing on demographic, clinical, and geographic trends. Objectives: To analyze dengue prevalence, demographic distribution, clinical symptoms, and serotype patterns in Bangladesh, with an emphasis on urban–rural disparities, gender differences, and serotype evolution. Methods: A systematic search was conducted across PubMed, Scopus, Web of Science, and Global Health (Ovid) databases, reviewing studies published from 2000 to 2024. Following PRISMA guidelines, 25 studies meeting eligibility criteria were selected. Data extraction and quality assessment were independently performed by three reviewers, ensuring methodological rigor. Results: Dengue incidence was higher in urban areas, mainly affecting males aged 20–34, with dengue virus serotype 3 (*DENV-3*) as the dominant serotype. Fever, headache, and joint pain were the most common symptoms, while severe cases often presented with respiratory and hemorrhagic complications. Acute symptoms like dyspnea and dehydration spread rapidly in densely populated areas. In rural areas, dengue showed a more endemic pattern, with persistent symptoms such as gastroenteritis and muscle pain. Conclusion: Dengue is now firmly endemic in Bangladesh, with clear geographic, demographic, and clinical differences. The dominance of *DENV-3* and its association with more severe illness highlight the need for targeted and context specific interventions. Control efforts should prioritize vector management, public education, and continuous surveillance in urban areas, while strengthening community surveillance and primary healthcare in rural settings. Further research on rural transmission and the clinical impact of *DENV-3* is essential to guide effective and tailored dengue control strategies.

## 1. Introduction

Dengue infection (DI) is a mosquito-borne arboviral disease transmitted through an infected female *Aedes* mosquito [1]. Dengue is characterized by fever and at least two symptoms such as headache, muscle and joint pain, rash, bleeding, and low white blood cell count. Diagnosis is confirmed through the detection of dengue antigen, antibodies, or viral RNA. *Dengue Infection* is caused by the four serotypes of the *Orthoflavivirus*, such as *DENV-1*, *DENV-2*, *DENV-3* and *DENV-4*, each capable of causing self-limiting fevers to severe conditions [2]. According to the 1997 WHO classification, dengue virus infections were classified into dengue fever (DF), dengue *hemorrhagic fever (DHF*), and *dengue shock syndrome (DSS)* [3]. According to the revised classification (WHO 2009), dengue is categorized based on the severity of their symptoms, including dengue without warning signs, dengue with warning signs (abdominal pain, persistent vomiting, fluid accumulation, mucosal bleeding, lethargy, liver enlargement, increasing hematocrit with decreasing platelets, and severe dengue [4,5].

Dengue is a major contributor of global morbidity and mortality [6,7]. Over the last 50 years the incidence of dengue has increased 30-fold globally [7,8]. The current estimates suggest that dengue affects over 390 million people annually, resulting in 500,000 hospitalizations and 25,000 deaths [9,10]. Dengue is now endemic in 129 countries, with the highest burden in South Asia, Southeast Asia, America, and the Western Pacific regions [11].

Dengue has emerged as a major public health concern in Bangladesh, where the significant outbreak was first reported in 2000. Studies indicate that dengue is now endemic in almost every district, extending to peri-urban and rural areas. The first reported dengue outbreak in Bangladesh occurred in 1960, known as “Dacca Fever” [7]. Since then, the country has experienced recurrent outbreaks, with cases reported nearly every year. Dengue has become increasingly common since 2000, with a clear upward trend in both incidence and severity [12,13]. The most severe epidemic to date occurred in 2023, resulting in a record-breaking 316,160 cases and 1643 dengue-related deaths [14]. In 2019, Bangladesh faced its second-largest dengue outbreak, with 101,354 cases and 164 deaths, marking a significant rise in both incidence and mortality [15,16]. In 2022, another substantial outbreak resulted in 60,078 cases and 266 deaths [17].

Dengue surveillance in Bangladesh has been established since 2000 under the Directorate General of Health Services through the Institute of Epidemiology Disease Control and Research [16,18]. Despite the increasing number of dengue related studies, the available evidence remains dispersed and heterogeneous across time periods, geographic settings, and population groups. Most of the studies report isolated findings on outbreaks, clinical features, or serotype circulation, which limits a coherent understanding of long-term epidemiological change. In particular, existing studies have not systematically synthesized evidence on temporal shifts in serotype dominance, urban and rural transmission dynamics, or gender specific risk patterns. The aim of this systematic review is to synthesize evidence published between 2000 and 2024 to characterize temporal trends in dengue epidemiology in Bangladesh, with a focus on demographic, clinical, virological, and geographic patterns. By consolidating more than two decades of evidence, this review provides a robust foundation to inform targeted and context specific public health strategies for dengue prevention and control.

Research questions:

1. What is the geographical distribution of dengue research across the divisional levels in Bangladesh?
2. What are the key epidemiological characteristics of dengue infection in Bangladesh?
3. Are there any gender disparities in the dengue infection?
4. Are there differences in dengue prevalence and incidence between urban and rural areas?
5. What are the most common clinical symptoms associated with dengue infection?
6. Which dengue serotypes are circulating in Bangladesh? Have there been any changes in the serotype distribution of dengue in Bangladesh over the past 20 years?

## 2. Methods

### 2.1. Protocol Registration

The systematic review protocol was registered in the International Prospective Register of Systematic Reviews—PROSPERO (Reg. No. CRD42023357093). We conducted a systematic review following the guidelines of Preferred Reporting Items for Systematic Reviews and Meta-Analyses (PRISMA) [19]. The PRISMA 2020 checklist is provided in the Supplementary File [Table S1].

### 2.2. Data Source and Search Strategy

The relevant articles were retrieved using PubMed, Scopus, Web of Science, and Global Health (Ovid) databases from 1 January 2000 to 31 December 2024. The initial systematic search was conducted in January 2023 and was subsequently updated in December 2024 using the same search strategy and eligibility criteria to ensure comprehensive capture of studies published during the intervening period. A combination of search terms was used, including dengue, DF, DHF, DSS, severe dengue, outbreaks, incidence, prevalence, mortality, risk factors, co-morbidities, environmental, socio-economic, ecological, demography, age, sex, ethnicity, gender, location, background, and climate change. Database-wise search strategies were presented in Supplementary File [Table S2]. The inclusion and exclusion criteria for the studies included in this review were shown in the Supplementary File [Table S3]. Details of excluded full-text studies and reasons for exclusion are provided in the Supplementary File [Table S4].

### 2.3. Study Selection

**Figure 1:**
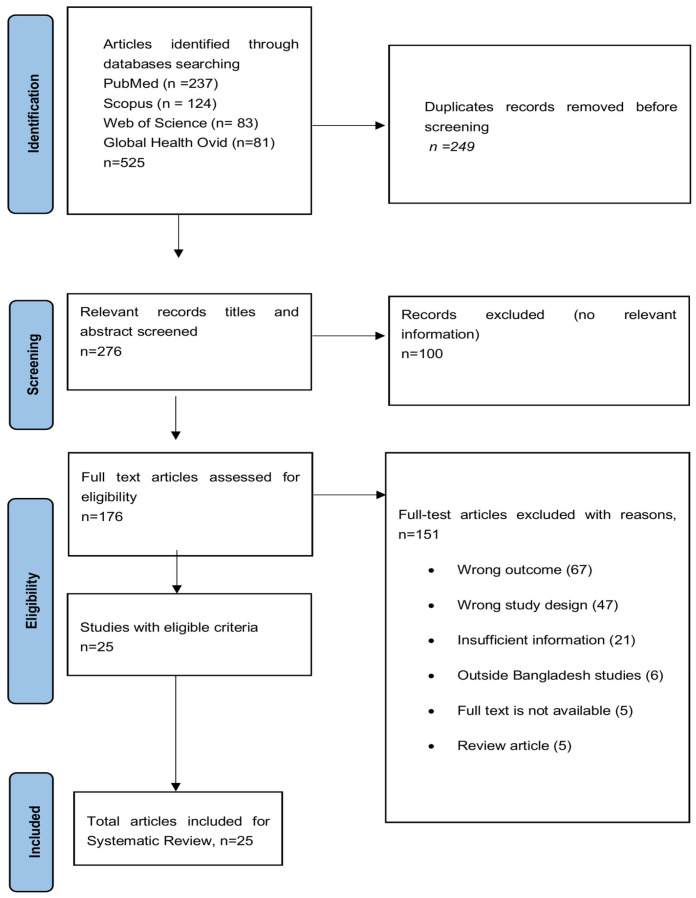
PRISMA flow diagram for the study selection.

All retrieved records were imported into EndNote 20 (Clarivate Analytics, Philadelphia, PA, USA) for reference management, and duplicate entries were removed. The remaining records were subsequently uploaded to Covidence (https://www.covidence.org/ accessed on 15 March 2023) for systematic screening. The initial search across PubMed, Scopus, Web of Science, and Global Health (Ovid) identified 525 articles. After removal of 249 duplicate records, 276 articles were screened based on titles and abstracts. Of these, 176 full text articles were assessed for eligibility. Following full-text review, 151 articles were excluded for predefined reasons, including irrelevant outcomes (*n* = 67), inappropriate study design (*n* = 47), insufficient information (*n* = 21), studies conducted outside Bangladesh (*n* = 6), unavailability of full text (*n* = 5), and review articles (*n* = 5). Ultimately, 25 studies met the inclusion criteria and were included in the final systematic review, as illustrated in Figure 1. The references of all 25 included studies are provided in the reference list.

### 2.4. Data Extraction and Analysis

The Microsoft excel were used for data extractions. Following information were included during the article extractions, such as, first author, location, study size, dengue infection, age, sex and clinical characteristics. Data extractions were performed by two independent reviewers (MK, SZH) using the same extraction sheet. Three independent reviewers were involved in the article screening and selection process. Two independent reviewers (MK and SZH) screened the titles and abstracts on Covidence based on the eligibility criteria. The same reviewers then assessed the full-text articles. Discrepancies were resolved by discussion with a third reviewer (TT).

### 2.5. Quality Assessment

The quality of the studies was assessed using the Newcastle–Ottawa Quality Assessment Scale (NOS) [20,21,22]. The NOS consists of eight points across three domains, such as selection, comparability and outcome. A numeric score was assigned for each criterion met, with a maximum score of eight, four for selection, two for comparability, and two for outcome. Higher scores indicate a lower risk of bias. The Agency of Healthcare Research standards were applied as the criterion for quality [21,23,24,25,26,27,28,29]. The quality of included cross-sectional, cohort, and case–control studies was assessed using the Newcastle–Ottawa Scale, with detailed scores provided in the Supplementary File [Tables S4–S6]. The full assessment criteria are presented in Supplementary Text S1. As with the screening process, quality assessment was conducted independently by two reviewers (MK and TT), and any discrepancies were resolved through discussion with a third reviewer (SZH) to achieve consensus.

## 3. Results

**Figure 2:**
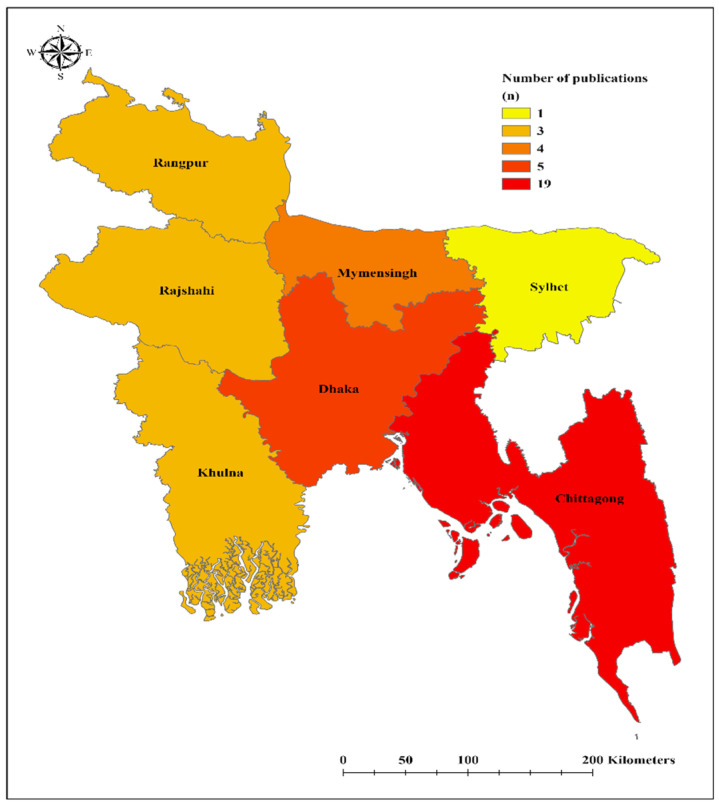
Regional distribution of 25 peer-reviewed published articles.

**Figure 3:**
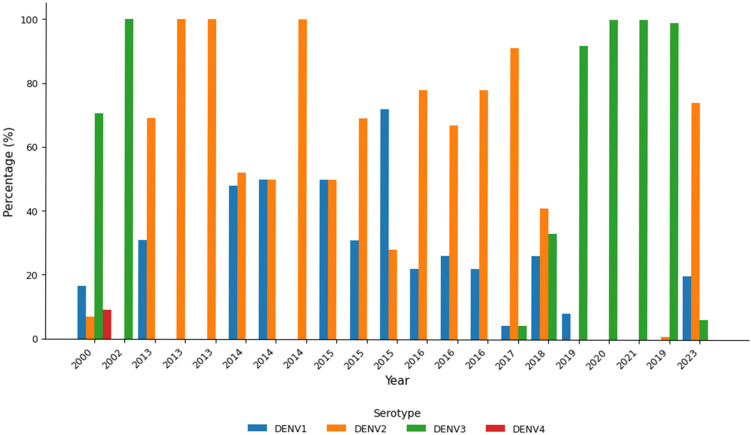
Circulation of dengue virus serotypes by its proportion in Bangladesh.

We first present a district-level map highlighting the regions where studies on dengue were conducted in Bangladesh [Figure 2]. Following this, we provide a summary of the demographic characteristics [Table S7], the clinical manifestations of dengue infection [Table S8], and the identified dengue serotypes [Figure 3]. Lastly, we offer an integrated discussion that combines the findings from the tables and figures.

Results for research question 1: the geographical distribution of dengue research across the divisional levels in Bangladesh. Figure 2 shows the distribution of 25 assessed peer-reviewed publications across the divisional level in Bangladesh. The Dhaka Division leads in research activity, with 17 publications, followed by Chittagong with five and Khulna with four studies. In contrast, Rajshahi, Barisal, and Sylhet each reported three studies, reflecting various research engagements.

Results based on research questions 2, 3 and 4: the key epidemiological characteristics of dengue infection in Bangladesh. Table S8 shows the demographic characteristics of dengue infection. The results from the 25 peer-reviewed articles show that the average age of participants ranges from seven years [30] to 34 years [31,32,33] suggesting that these studies address different population segments. The majority of these study participants were over 20 years old [31,34,35,36,37,38,39,40,41], only a small proportion were aged below 10 years old [30]. Gender distribution shows that most of the study participants were male, with the proportion ranging from 66.6% [40] to as high as 70% [38,42]. A wide variation was noted in terms of spatial distribution, showing strong urban concentration of cases. The proportion of cases in urban areas ranged from 87% [35] to 100% [37,39]. However, one study reported more cases in rural areas, with 76.4% [43] of cases in rural settings. The studies included in this analysis also examined whether dengue cases were related to endemic transmission or specific outbreaks. Among those endemic and outbreak events, 65% [30,31,33,38,40,41,42,44,45,46,47,48,49,50] published articles focused on endemic conditions.

Table S9 summarizes the severe and common clinical manifestation profiles of dengue patients. Among common symptoms, fever was reported in 96.6% to 100% across all studies [31,35,36,37,38,39,40,44,45,51,52]. Headache shows significant variability, ranging from 21.0% [52] to 96.0% [31]. Cough was less frequently reported, with an incidence ranging from 2.8% [38] to 39.7% [35]. Gastrointestinal symptoms such as nausea and vomiting were prominent in several studies, with nausea/vomiting reported in 32.0% to 93.0% of cases [31,35,36,37,38,40,49,51,52]. There are fewer reports of respiratory symptoms, such as respiratory distress [31,35,36,37,38,39,40,44,51,52]. Pain-related symptoms such as body ache and joint pain also vary, with body ache noted in 26.6% to 57.7% [35,51] and joint pain (arthralgia) in 4.5% to 40.8% [31,36]. In contrast, severe clinical manifestations were less frequently reported. Hemorrhagic features, such as bleeding and hematuria, were uncommon overall but notable in some studies [36,43,44,49,51,52]. Respiratory complications, including respiratory distress, were also frequently reported with an incidence ranging from 1.5% to 15.5% of cases [35,36,49].

Figure 3 illustrates the distribution of dengue virus serotypes in Bangladesh from 2000 to 2021. The color of the bar corresponds to each serotype—*DENV-1* (blue), *DENV-2* (orange), *DENV-3* (green), and *DENV-4* (red), and the height correspond to the proportion (%) of each serotype. *DENV-3* was predominant in 2000 and 2002, re-emerged in 2017 and was the most dominant serotype since 2019 *DENV-1* and *DENV-2* had periodic peaks, especially in 2014 and 2015. *DENV-4* appeared infrequently. In 2023, *DENV-2* emerged as the predominant serotype (74.1%), followed by *DENV-1* (19.8%) and *DENV-3* (6.1%), indicating a marked shift from the *DENV-3* predominance observed in preceding years. Its highest percentage was in 2000, with minimal presence thereafter. Overall, this chart highlights changes in serotype prevalence over time. It showed the changing nature of dengue virus circulation in Bangladesh.

We integrated demographic data from Table S7 with clinical findings from Table S8. This analysis showed differences in dengue exposure and symptoms across regions and populations. Studies found higher numbers of affected individuals in urban areas [35,52]. They showed that acute symptoms such as dyspnea and dehydration spread rapidly in densely populated areas. A study observed a more endemic pattern in rural areas, with persistent symptoms like gastroenteritis and muscle pain [31]. Another study recorded high rates of severe symptoms, such as dehydration and respiratory distress [35]. Relationships between gender, environment, and symptom severity were also noted, with males and urban residents more likely to experience severe symptoms [40]. Fever and joint pain were widespread in both urban and rural settings, highlighting dengue’s significant impact on public health. These findings suggested a need for targeted public health interventions to address outbreaks and endemic spread.

## 4. Discussion

This systematic review identifies several key patterns in dengue epidemiology in Bangladesh, including geographic disparities, demographic vulnerabilities, symptom variation, and a shift in the dominant serotype. Overall, the evidence suggests that dengue burden and presentation are influenced by a combination of population density, environmental conditions favorable to *Aedes* mosquitoes, healthcare access, and evolving viral circulation. Most studies were conducted in urban settings, with comparatively limited evidence from rural regions, which may influence the representation of national patterns. The higher reported prevalence among males and the affected age range of seven to 34 years indicates age and gender vulnerabilities that are likely driven by exposure and context rather than biological susceptibility alone. Urban studies frequently report acute symptoms, particularly during outbreaks, which is consistent with rapid transmission in densely populated environments. In contrast, rural cases appear to persist for longer and may present with gastroenteritis and muscle pain, possibly reflecting delayed diagnosis, limited access to care, or differences in transmission patterns. Clinical manifestations vary widely, with high fever, headache, and joint pain commonly reported, alongside less frequent but clinically important respiratory and hemorrhagic features. Notably, this review indicates that DENV-3 has become the dominant serotype, signaling a meaningful epidemiological shift in Bangladesh with potential implications for disease severity and control priorities.

Our review shows that dengue research activity is heavily concentrated in the Dhaka region. The high volume of studies from Dhaka likely reflects its dense population, where outbreaks are more frequent and severe, as well as its comparatively strong surveillance infrastructure, which enables rapid outbreak detection and timely data collection. In contrast, regions with limited research capacity may experience delays in case identification and management, contributing to poorer health outcomes and underrepresentation in the literature. Similar concentrations of research in major urban centers have been reported in other settings, where surveillance and reporting systems are more developed than in peripheral or rural areas [53]. This comparison indicates that the apparent geographical distribution of dengue research in Bangladesh may partly reflect variations in surveillance capacity and data availability rather than the true disease burden alone. While the concentration of studies in Dhaka strengthens evidence on urban transmission dynamics, it also reveals an important limitation: national estimates might be biased if areas with weaker surveillance remain under-studied. Policy efforts should therefore promote more balanced research coverage and enhance surveillance beyond major cities to support equitable and evidence-based dengue control across the country.

Our research highlights significant disparities in dengue infection rates between urban and rural areas in Bangladesh, with urban settings experiencing notably higher incidence. This difference may be attributed to high population density in cities, which facilitates mosquito breeding and transmission, together with rapid urbanization that creates favorable environments for *Aedes* mosquitoes. For example, cities such as Dhaka face frequent dengue outbreaks due to inadequate waste management and the presence of stagnant water sources that serve as breeding sites. Part of the observed disparity may also reflect better surveillance and reporting systems in Dhaka compared with rural and remote areas, where cases may be under-detected. Similar urban–rural patterns have been reported in other countries, including Thailand and India, where healthcare access and vector control are often less effective in rural settings [54,55]. These findings indicate that public health interventions must be tailored to the specific demographic and environmental conditions of each area. Bangladesh may therefore require a balanced approach to dengue control, with equitable research funding and targeted interventions and tailored to the distinct needs of both urban and rural regions to reduce overall infection rates.

In Bangladesh, males are more affected by dengue infection than females. This is likely due to men’s increased involvement in outdoor activities, which heightens their risk of mosquito bites. Additionally, men may be less inclined to use protective measures, such as wearing long-sleeved clothing or applying mosquito repellent, further increasing their risk of infection. Similar findings have been observed in other dengue-endemic regions, including Singapore, Thailand, Bhutan, and India, where studies report that men face a higher risk of infection due to occupational exposure. In Bangladesh, dengue is reported more often among males than females, but this pattern should be interpreted cautiously. The difference probably does not reflect biological susceptibility but is more likely related to differing exposure levels. Men are more frequently involved in outdoor and mobile work, which raises contact with *Aedes* mosquitoes in occupational and public environments. Variations in healthcare-seeking behavior and access to diagnosis may also lead to under-detection among women. Such trends are similarly noted in other dengue-endemic nations, where higher male incidence correlates with occupational exposure and daily movement patterns. These gender disparities in dengue infection are mainly influenced by social and environmental factors rather than inherent biological risks. An important implication is that surveillance data might underestimate the true burden of disease among women if access to healthcare and diagnosis is unequal. Therefore, dengue control strategies in Bangladesh should adopt gender-sensitive approaches, including targeted protection for outdoor workers and enhanced access to diagnosis and care for both men and women. These consistent patterns suggest that public health strategies in Bangladesh could benefit from gender-specific interventions to address exposure differences. Such program’s might include promoting protective clothing and mosquito repellent use among outdoor workers to help reduce dengue transmission across all regions.

Fever was identified as the most common symptom of dengue infection, with 96.6% to 100% of patients presenting with fever, confirming its value as a key clinical marker for diagnosis. This high prevalence likely reflects the host immune response to viral infection and the high transmission intensity associated with Bangladesh’s tropical climate, which supports mosquito breeding. This pattern is consistent with evidence from Southeast Asia, South America, Africa, and the Caribbean, where fever has been reported in approximately 98% of more than 50,000 dengue cases worldwide [56]. The consistency of fever across diverse geographic settings underscores its importance for early screening and clinical suspicion. However, our review also revealed substantial variability in other symptoms, including headache and gastrointestinal manifestations. Previous studies have reported headache prevalence ranging from 20% in South America to nearly 95% in Southeast Asia [57]. This contrast highlights the heterogeneous clinical presentation of dengue and suggests that rigid symptom-based case definitions may fail to capture atypical presentations. These findings support the need for flexible diagnostic approaches that account for regional variation in symptom profiles, as well as standardized clinical guidelines that prioritize fever while recognizing the broader spectrum of dengue manifestations to improve timely diagnosis and patient management.

This review indicates that DENV-3 has become the dominant serotype in Bangladesh and is strongly linked to severe disease. The rise in severe DENV-3 cases is probably related to high rates of secondary infection, where individuals previously exposed to a different serotype face a higher risk of adverse outcomes. One plausible explanation is the limited circulation of DENV-3 between 2013 and 2016, which may have decreased population immunity and heightened susceptibility when the serotype re-emerged. Similar patterns have been observed in Brazil, Thailand, and India, where shifts in serotype dominance are associated with increased disease severity in individuals with prior exposure [58,59,60]. This comparison suggests that changes in viral circulation can significantly impact clinical outcomes on a population level. Conversely, DENV-1, while also common, is generally linked to milder illness and tends to affect individuals without prior dengue exposure. Evidence from Sri Lanka and India supports the idea that DENV-1 usually causes less severe disease in dengue-naïve populations. These findings highlight that serotype-specific dynamics should shape control strategies: while DENV-3 demands enhanced surveillance, targeted vaccination, and public awareness of secondary infection risks, controlling DENV-1 may be more effectively achieved through community-based vector control and education to reduce transmission in densely populated areas.

Our findings show that about 57% of dengue research in Bangladesh focuses on endemic conditions, while the rest examines outbreaks. This pattern indicates an increasing acknowledgement of dengue as a constant public health issue that requires ongoing management rather than sporadic responses. Endemic transmission is probably sustained by year-round climatic conditions conducive to mosquito breeding and high population density, which together facilitate continuous virus circulation. This aligns with evidence from other dengue-endemic areas, including Southeast Asia and Brazil, where long-term management is prioritized due to consistent transmission between outbreaks.

This systematic review was undertaken to synthesize the epidemiological, demographic, clinical, and virological evidence on dengue in Bangladesh and to clarify how transmission patterns, disease presentation, and serotype circulation have evolved over time. By integrating findings across geographic settings, population groups, and clinical outcomes, this study provides a comprehensive overview of how dengue burden in Bangladesh is shaped by environmental context, exposure-related vulnerability, and changes in viral dynamics. The most important contribution of this review is the identification of a sustained shift towards DENV-3 dominance, together with evidence that transmission is increasingly influenced by urbanization, occupational exposure, and persistent endemic circulation rather than isolated outbreaks alone. These findings demonstrate that dengue in Bangladesh represents a long-term public health challenge that requires continuous, context-specific control strategies.

Despite these contributions, several key questions remain unanswered. To what degree do environmental and climatic factors, such as temperature, rainfall, humidity, and urban growth, influence regional and seasonal variation in transmission? How much of the urban–rural gap is due to real differences in incidence versus under-detection in areas with limited surveillance and healthcare access? What mechanisms explain the increasing prevalence of DENV-3, and how does previous serotype exposure affect population vulnerability to severe disease? Answering these questions will require long-term studies that integrate epidemiological surveillance with entomological, environmental, and health-system data. This work is critical for improving early warning systems, refining risk assessment, and creating sustainable, evidence-based dengue control strategies in Bangladesh and other endemic regions.

### 4.1. Strengths and Limitations

This study provides a comprehensive synthesis of dengue epidemiology in Bangladesh and underscores the importance of these findings for public health planning. Following PRISMA guidelines and registering with PROSPERO ensured transparency in the methodology. Including studies from over two decades enabled examination of long-term epidemiological trends, and searching multiple databases reduced the risk of bias associated with specific sources. Several limitations must be acknowledged. Restricting to English-language publications and excluding gray literature may have introduced publication bias and geographic selection bias. Variability in study design, data quality, and population coverage across the included studies may also affect comparability. The predominance of male participants in several studies may affect the generalisability of the results. Most notably, many studies were hospital or clinic-based and did not report environmental or meteorological variables such as temperature, rainfall, humidity, or land use. Consequently, this review could not evaluate the impact of climate or environmental factors on dengue risk. Differences in surveillance and reporting between urban and rural areas may also have contributed to underestimation in resource-limited settings.

### 4.2. Future Research

Future research should incorporate more studies from rural areas of Bangladesh. This will better capture regional differences in dengue transmission and access to healthcare. Greater attention to gender-related vulnerabilities is necessary. Occupational exposure among males should be examined to explain higher infection rates. Further investigation of DENV 3 and its association with severe secondary infections is crucial. Future studies should also employ long-term integrated approaches. Dengue surveillance must be linked with environmental and climate indicators, including temperature, rainfall, humidity, and urban expansion. Epidemiological data should be combined with entomological and geographic methods. This will enhance understanding of climate change, urbanization, and transmission patterns. Such evidence is vital for early warning systems and will support targeted, sustainable dengue control strategies.

## 5. Conclusions

This systematic review summarizes key epidemiological patterns of dengue in Bangladesh and identifies significant geographic, demographic, and clinical differences. Dengue remains more common in urban areas, reflecting population density and infrastructure-related challenges. Rural areas exhibit more persistent and under-recognized disease patterns. The dominance of DENV-3, which is associated with more severe illness, indicates a need for targeted intervention. The higher burden among males, likely due to occupational exposure, highlights the importance of gender-responsive prevention.

Effective dengue control in Bangladesh requires a context-specific approach. In urban areas, priorities include targeted vector control, source reduction in high rise buildings, improved waste management, and urban planning to reduce breeding sites. In rural areas, key actions are strengthening community-based surveillance, improving primary healthcare capacity, and expanding mobile health services. These measures should be implemented through existing national systems led by the Directorate General of Health Services and the Institute of Epidemiology, Disease Control and Research. Long-term control will require a holistic, multisectoral strategy involving the Ministry of Education, the Ministry of Local Government, and the Ministry of Religious Affairs, supported by a unified action plan and sustained public awareness programs.

## Data Availability

The datasets used and/or analyzed during the current study are included in this published article and the Supplementary File. All of these are also available in the public domain.

## Supplementary Materials

The following supporting information can be downloaded at https://www.mdpi.com/article/10.3390/ijerph23020235/s1, Table S1: PRISMA 2020 Checklist; Table S2: Search terms used to identify records from databases; Table S3: Eligibility criteria for selecting the studies; Table S4: Full-Text Articles Excluded and Reasons for Exclusion; Table S5: Quality Assessment Scores for Cross-Sectional Studies Based on the Newcastle–Ottawa Scale; Table S6: Quality Assessment of Retrospective Cohort Studies Using the Newcastle–Ottawa Scale; Table S7: Quality Assessment of Case–Control Studies Using the Newcastle–Ottawa Scale; Table S8: Demographic characteristics in the included studies; Table S9: Clinical profiles of dengue patients; Text S1: Newcastle–Ottawa Scale criteria.

## Abbreviations

PRISMA	Preferred Reporting Items for Systematic Reviews and Meta-Analyses
WHO	World Health Organization
CVD	Cardiovascular diseases
DENV	Dengue virus
DI	Dengue Infection
RNA	Ribonucleic acid
SD	Severe dengue
DF	Dengue fever
DHF	Dengue hemorrhagic fever
DSS	Dengue shock syndrome
