# Supplementary material for: Epidemiological Characteristics of Dengue Infection in Bangladesh: A Systematic Review
Source: Int J Environ Res Public Health. 2026 Feb 13;23(2):235. doi: 10.3390/ijerph23020235 (PMC12940295; doi:10.3390/ijerph23020235)
Supplement: Supplementary file 1 [file ijerph-23-00235-s001.zip › ijerph-3962138-supplementary.pdf]

Epidemiological Characteristics of Dengue Infection in Bangladesh: A Systematic Review

Table S1 PRISMA 2020 Checklist

Section and Topic	Item #	Checklist item	Location where item is reported
**TITLE**			
Title	1	Identify the report as a systematic review.	Page 1
**ABSTRACT**			
Abstract	2	See the PRISMA 2020 for Abstracts checklist.	Page 1
**INTRODUCTION**			
Rationale	3	Describe the rationale for the review in the context of existing knowledge.	Pages 2–3
Objectives	4	Provide an explicit statement of the objective(s) or question(s) the review addresses.	Pages 3
**METHODS**			
Eligibility criteria	5	Specify the inclusion and exclusion criteria for the review and how studies were grouped for the syntheses.	Page 3 (Methods 2.2)
Information sources	6	Specify all s, registers, websites, organisations, reference lists and other sources searched or consulted to identify studies. Specify the date when each source was last searched or consulted.	Page 3 (Methods 2.2)
Search strategy	7	Present the full search strategies for all databases, registers and websites, including any filters and limits used.	Page 3 + Supplementary Table S2
Selection process	8	Specify the methods used to decide whether a study met the inclusion criteria of the review, including how many reviewers screened each record and each report retrieved, whether they worked independently, and if applicable, details of automation tools used in the process.	Page 3–4 (Methods 2.3)
Data collection process	9	Specify the methods used to collect data from reports, including how many reviewers collected data from each report, whether they worked independently, any processes for obtaining or confirming data from study investigators, and if applicable, details of automation tools used in the process.	Page 4 (Methods 2.4)
Data items	10a	List and define all outcomes for which data were sought. Specify whether all results that were compatible with each outcome domain in each study were sought (e.g. for all measures, time points, analyses), and if not, the methods used to decide which results to collect.	Page 4 (Methods 2.4)
	10b	List and define all other variables for which data were sought (e.g. participant and intervention characteristics, funding	

Section and Topic	Item #	Checklist item	Location where item is reported
		sources). Describe any assumptions made about any missing or unclear information.	**Page 4 (Methods 2.4)**
Study risk of bias assessment	11	Specify the methods used to assess risk of bias in the included studies, including details of the tool(s) used, how many reviewers assessed each study and whether they worked independently, and if applicable, details of automation tools used in the process.	**Page 4 (Methods 2.5)**
Effect measures	12	Specify for each outcome the effect measure(s) (e.g. risk ratio, mean difference) used in the synthesis or presentation of results.	Not applicable (descriptive synthesis)
Synthesis methods	13a	Describe the processes used to decide which studies were eligible for each synthesis (e.g. tabulating the study intervention characteristics and comparing against the planned groups for each synthesis (item #5)).	Page 4 (Methods 2.4–2.5)
	13b	Describe any methods required to prepare the data for presentation or synthesis, such as handling of missing summary statistics, or data conversions.	Page 4 (Methods 2.4)
	13c	Describe any methods used to tabulate or visually display results of individual studies and syntheses.	Page 4 (Methods 2.4)
	13d	Describe any methods used to synthesize results and provide a rationale for the choice(s). If meta-analysis was performed, describe the model(s), method(s) to identify the presence and extent of statistical heterogeneity, and software package(s) used.	Page 4 (Methods 2.4)
	13e	Describe any methods used to explore possible causes of heterogeneity among study results (e.g. subgroup analysis, meta-regression).	Not applicable (no meta-analysis)
	13f	Describe any sensitivity analyses conducted to assess robustness of the synthesized results.	Not applicable
Reporting bias assessment	14	Describe any methods used to assess risk of bias due to missing results in a synthesis (arising from reporting biases).	Not assessed / Not applicable
Certainty assessment	15	Describe any methods used to assess certainty (or confidence) in the body of evidence for an outcome.	Not assessed / Not applicable
**RESULTS**			
Study selection	16a	Describe the results of the search and selection process, from the number of records identified in the search to the number of studies included in the review, ideally using a flow diagram.	Page 4–5 (Results + Figure 1)
	16b	Cite studies that might appear to meet the inclusion criteria, but which were excluded, and explain why they were excluded.	Page 4 + Supplementary Table S4
Study characteristics	17	Cite each included study and present its characteristics.	Page 5 + Table s7
Risk of bias in studies	18	Present assessments of risk of bias for each included study.	Page 5 + Supplementary Tables S5–S7

Section and Topic	Item #	Checklist item	Location where item is reported
Results of individual studies	19	For all outcomes, present, for each study: (a) summary statistics for each group (where appropriate) and (b) an effect estimate and its precision (e.g. confidence/credible interval), ideally using structured tables or plots.	Pages 5–6 (Tables S7–S9, Figures 2–3)
Results of syntheses	20a	For each synthesis, briefly summarise the characteristics and risk of bias among contributing studies.	Page 6 (Results narrative)
	20b	Present results of all statistical syntheses conducted. If meta-analysis was done, present for each the summary estimate and its precision (e.g. confidence/credible interval) and measures of statistical heterogeneity. If comparing groups, describe the direction of the effect.	Page 6 (Results narrative)
	20c	Present results of all investigations of possible causes of heterogeneity among study results.	Not applicable
	20d	Present results of all sensitivity analyses conducted to assess the robustness of the synthesized results.	Not applicable
Reporting biases	21	Present assessments of risk of bias due to missing results (arising from reporting biases) for each synthesis assessed.	Not assessed / Not applicable
Certainty of evidence	22	Present assessments of certainty (or confidence) in the body of evidence for each outcome assessed.	
**DISCUSSION**			
Discussion	23a	Provide a general interpretation of the results in the context of other evidence.	Pages 6–7 (Discussion)
	23b	Discuss any limitations of the evidence included in the review.	Page 7 (Strengths and Limitations)
	23c	Discuss any limitations of the review processes used.	Page 7 (Strengths and Limitations)
	23d	Discuss implications of the results for practice, policy, and future research.	Pages 6–7 (Discussion & Conclusions)
**OTHER INFORMATION**			
Registration and protocol	24a	Provide registration information for the review, including register name and registration number, or state that the review was not registered.	Page 3 (Methods 2.1)
	24b	Indicate where the review protocol can be accessed, or state that a protocol was not prepared.	Page 3 (Methods 2.1)
	24c	Describe and explain any amendments to information provided at registration or in the protocol.	Not applicable
Support	25	Describe sources of financial or non-financial support for the review, and the role of the funders or sponsors in the review.	Page 7 (Funding)
Competing interests	26	Declare any competing interests of review authors.	Page 7 (Conflicts of Interest)
Availability of data, code	27	Report which of the following are publicly available and where they can be found: template data collection forms; data extracted from included studies; data used for all	**Page 7 (Data Availability Statement)**

Section and Topic	Item #	Checklist item	Location where item is reported
and other materials		analyses; analytic code; any other materials used in the review.	

Table S2 Search terms used to identify records from databases (Last search done on Thursday, 31/12/2024)

**PubMed**<br>(Dengue OR "Dengue fever" OR "Dengue haemorrhagic fever" OR "Dengue shock syndrome" OR "Severe dengue" OR DENV)) AND ((incidence OR prevalence OR Mortality OR "Risk factors" OR "Cost of illness" OR "Economic burden" OR "Burden of disease" OR DALY)) AND ((outbreak OR Co-morbidities OR environmental OR "Social class" OR Socio-economic OR Ecological OR Demography OR Age factors OR Sex OR "Ethnic group" OR Gender OR gender OR location OR Background OR location OR resident OR nation OR "Climate change")) AND (Bangladesh)) Filters: Abstract, from 2000/1/1 - 2024/12/31
**Scopus**<br>(TITLE-ABS-KEY (Bangladesh)) AND (TITLE-ABS-KEY (outbreak OR co-morbidities OR environmental OR "Social class" OR socio-economic OR ecological OR demography OR age AND factors OR sex OR "Ethnic group" OR gender OR gender OR location OR background OR location OR resident OR nation OR "Climate change")) AND (TITLE-ABS-KEY (incidence OR prevalence OR mortality OR "Risk factors" OR "Cost of illness" OR "Economic burden" OR "Burden of disease" OR daly)) AND (TITLE-ABS-KEY (dengue OR "Dengue fever" OR "Dengue haemorrhagic fever" OR "Dengue shock syndrome" OR "Severe dengue" OR denv)) AND PUBYEAR > 2001 AND PUBYEAR < 2025 (2000/1/1 - 2024/12/31)
**Web of science**<br>(((TS=(Bangladesh)) AND TS=(Dengue OR "Dengue fever" OR "Dengue haemorrhagic fever" OR “Dengue shock syndrome” OR “Severe dengue” OR DENV)) AND TS=(incidence OR prevalence OR Mortality OR “Risk factors” OR “Cost of illness” OR “Economic burden” OR “Burden of disease” OR DALY)) AND TS=(outbreak OR Co-morbidities OR environmental OR “Social class” OR Socio-economic OR Ecological OR Demography OR Age factors OR Sex OR “Ethnic group” OR Gender OR gender OR location OR Background OR location OR resident OR nation OR "Climate change") 2025 (2024/1/1 - 2024/12/31)
**Global Health (Ovid)**

(Dengue OR "Dengue fever" OR "Dengue haemorrhagic fever" OR "Dengue shock syndrome" OR "Severe dengue" OR DENV)) AND (incidence OR prevalence OR Mortality OR "Risk factors" OR "Cost of illness" OR "Economic burden" OR "Burden of disease" OR DALY)) AND (outbreak OR Co-morbidities OR environmental OR "Social class" OR Socio-economic OR Ecological OR Demography OR Age factors OR Sex OR "Ethnic group" OR Gender OR gender OR location OR Background OR location OR resident OR nation OR "Climate change")) AND (Bangladesh)) Filters: Abstract, from 2000/1/1 - 2024/12/31

Table S3 Eligibility criteria for selecting the studies.

Inclusion criteria	Exclusion Criteria
Studies on human dengue infections in Bangladesh	Studies from countries other than Bangladesh or on non-human infections.
Research conducted from the year between 2000 - 2024	Articles published before 2000
Articles published in English that report on dengue virus infections.	Non-English articles.
Observational studies, including cohort, cross-sectional, and studies on the epidemiology of dengue and its risk factors	Studies lacking extractable primary data or involving non-native individuals traveling from other countries.
Studies covering different strains of the dengue virus including DENV1, DENV2, DENV3, and DENV4	Case reports, case series, reviews, letters, grey literature, information from local newspapers and unpublished articles or conference papers.

Table S4 Full-Text Articles Excluded and Reasons for Exclusion

Authors	Article names	Reason for exclusion
2. Abir et al, 2020	Dengue in Dhaka, Bangladesh: Hospital-based cross-sectional KAP assessment at Dhaka North and Dhaka South City Corporation area	Exclude
6. Al-Amin et al. 2023	Insecticide resistance compromises the control of Aedes aegypti in Bangladesh	Wrong outcome
8 - Alam 2004	Management of dengue by the WHO guided national guidelines	Wrong outcome and wrong study design
9. WAGATSUMA et al. 2003	use of a geographic information system for defining spatial risk for dengue transmission in bangladesh: role for aedes albopictus in an urban outbreak	Wrong study design

#14 - Aziz 2002	Predominance of the DEN-3 genotype during the recent dengue outbreak in Bangladesh	Full article is not available and Wrong outcome
20. Banu et al. 2012	Space-time clusters of dengue fever in Bangladesh	Wrong outcome
27 - Chowdhury 2004	Seroprevalence of dengu infections amongst the children	Full article is not available
33. Das et al. 2021	Knowledge, attitude and prevention practices of garment factory workers regarding the largest Dengue outbreak on record in Bangladesh	Wrong outcome
36. Dey et al. 2022	Prediction of dengue incidents using hospitalized patients, metrological and socioeconomic data in Bangladesh: A machine learning approach	Wrong outcome and study design
39. Dhar-Chowdhury et al. 2016	Dengue Disease Risk Mental Models in the City of Dhaka, Bangladesh: Juxtapositions and Gaps Between the Public and Experts	Wrong outcome
47. Ferdous et al. 2016	Identification of Essential Containers for Aedes Larval Breeding to Control Dengue in Dhaka, Bangladesh	Wrong outcome
50. Haider et al. 2021	Dengue outbreaks in Bangladesh: Historic epidemic patterns suggest earlier mosquito control intervention in the transmission season could reduce the monthly growth factor and extent of epidemics	Insufficient information
61. Hossain et al. 2021	Knowledge, awareness and preventive practices of dengue outbreak in Bangladesh: A countrywide study	Insufficient information
72. Islam S et al. 2020	Association among ecological and behavioural attributes, dengue vector and disease control- a cross-sectional study of the city of Dhaka	Insufficient information

80. Kamal et al.2023	Relationship between Urban Environmental Components and Dengue Prevalence in Dhaka City-An Approach of Spatial Analysis of Satellite	Wrong outcome
84. Kayesh MEH et al. 2023	Increasing Dengue Burden and Severe Dengue Risk in Bangladesh- An Overview	Insufficient information
93. LaRocque et al.2021	Leptospirosis during Dengue Outbreak, Bangladesh	Wrong outcome
99. Mahmud et al. 2021	Megacities as drivers of national outbreaks: The 2017 chikungunya outbreak in Dhaka, Bangladesh	Wrong outcome
101. Maude RR et al. 2016.	BD, Chittagong.A prospective study of the importance of enteric fever as a cause of non-malarial febrile illness in patients admitted to Chittagong Medical College Hospital, Bangladesh	Wrong outcome<br>Wrong study design
106. Morales et al. 2016	Seasonal Distribution and Climatic Correlates of Dengue Disease in Dhaka, Bangladesh	Wrong outcome
110. Mutsuddy p et al. 2019	Dengue Situation in Bangladesh- An Epidemiological Shift in terms of Morbidity and Mortality	Insufficient information
113. Naher S. et al. 2022	Forecasting the incidence of dengue in Bangladesh-Application of time series model	Wrong outcome and study design
118. Paul et al. 2018.	Risk factors for the presence of dengue vector mosquitoes, and determinants of their prevalence and larval site selection in Dhaka, Bangladesh	Wrong outcome
120 – Pervin et al. 2002	Isolation and serotyping of dengue viruses by mosquito inoculation technique from clinically suspected cases of dengue fever	Wrong outcome
122. Podder 2006	Origin of dengue type 3 viruses associated with the dengue outbreak in Dhaka, Bangladesh, in 2000 and 2001	Review article
129. Rahim MA et al. 2018	Chikungunya-dengue co-infection during pregnancy requiring preterm Caesarean section-first case report from Bangladesh	Insufficient information and

		unclear case definition
130. Rahman 2020	An Association between Rainy Days with Clinical Dengue Fever in Dhaka, Bangladesh- Findings from a Hospital Based Study	Wrong outcome
135. Rahman MM et al. 2022	Dengue Fever Responses in Dhaka City, Bangladesh- A Cross-Sectional Survey	Wrong outcome
#142 - Rahman 2007	Seropositivity and pattern of dengue infection in Dhaka city	Full review is not available and Insufficient information
143. Rahman et al. 2022.	Dengue in Bangladesh: Strategic Assessment Considering the Future Outbreak and Hospital Scenario	Wrong setting
145. Riad et al. 2021.	Risk Assessment of Dengue Transmission in Bangladesh Using a Spatiotemporal	Wrong outcome and Wrong setting
150 - Salma 2021	Sociodemographic and Clinico-laboratory Profile of Expanded Dengue Syndrome: Experience from a Tertiary Hospital of Dhaka, Bangladesh	Full review is not available
151. Sarder et al 2022	Predicting Dengue Outbreak from Climate Data Using Machine Learning Algorithms	Wrong outcome and wrong study design
153. Sarker MMR et al. 2021.	Dengue Fever- Therapeutic Potential of Carica papaya L. Leaves	Outside Bangladesh studies
161. Sharmin 2013	Dengue infection in Dhaka City, Bangladesh	
162. Sharmin et al. 2015	Interaction of Mean Temperature and Daily Fluctuation Influences Dengue Incidence in Dhaka, Bangladesh	Wrong outcome
163. Sharmin et al. 2018.	BD. Geostatistical mapping of the seasonal spread of under-reported dengue cases in Bangladesh	Wrong outcome
164. Sharmin et al..2018	A Bayesian approach for estimating under-reported dengue incidence with a focus on non-	Wrong outcome

	linear associations between climate and dengue in Dhaka, Bangladesh	
#165 - Sharmin 2015	The emergence of dengue in Bangladesh: epidemiology, challenges and future disease risk	Wrong study design
171. Sultana A et al. 2020	Renal Involvement in Children with Dengue Fever- A Study in Tertiary Care Hospital of Bangladesh	Wrong outcome<br>And wrong setting
174. Tasnim et al. 2021. BD.	Observing the Unobserved: A Newspaper Based Dengue Surveillance System for<br>the Low-Income Regions of Bangladesh	Wrong outcome
181 - Uddin 2014	Clinico-pathological profile of dengue syndrome: an experience in a tertiary care hospital, Dhaka, Bangladesh	Full review is not available and Insufficient information
184. Wagatsuma et al. 2004	Dengue fever outbreak in a recreation club, Dhaka, Bangladesh	Insufficient case information and Wrong study design
187. Yang et al. 2023.	Demographic characteristics, clinical symptoms, biochemical markers and probability of occurrence of severe dengue: A multi-centre hospital-based study in<br>Bangladesh	Wrong Study design
189. Zahirul Islam et al. 2018	Correlates of Climate Variability and Dengue Fever in Two Metropolitan Cities in Bangladesh	Insufficient information
#225 - Ali 2024	**The recent burden of dengue infection in Bangladesh: A serious public health issue**	Insufficient Information
#225 - Ali 2024	**The recent burden of dengue infection in Bangladesh: A serious public health issue**	Wrong Study Design
#273 - Hasan 2024	**Two decades of endemic dengue in Bangladesh (2000-2022): trends, seasonality, and impact of**	Wrong Outcome

	**temperature and rainfall patterns on transmission dynamics.**	
#365 - Hossain 2024	**Spatio-temporal patterns of dengue in Bangladesh during 2019 to 2023: implications for targeted control strategies.**	Wrong outcome
#224 - Hossain 2024	**Community engagement and social participation in dengue prevention: A cross-sectional study in Dhaka City**	Insufficient Information
#244 - Islam 2024	**Rapid human movement and dengue transmission in Bangladesh: a spatial and temporal analysis based on different policy measures of COVID-19 pandemic and Eid festival.**	Wrong study design
#262 - Khan 2024	**Bangladesh records persistently increased number of dengue deaths in recent years: Dissecting the shortcomings and means to resolve.**	Wrong outcome
#227 - Mahmud 2024	**Alarming Trends in Dengue Incidence and Mortality in Bangladesh**	Wrong outcome
#229 - MehediHasanSumon 2024	**Understanding dengue outbreaks in Rajshahi district, Bangladesh: A comprehensive case study**	Review reports (Case)
#221 - Miah 2024	**Assessing the impact of climatic factors on dengue fever transmission in Bangladesh**	Wrong outcome
#225 Ahammad 2024		Wrong outcome

	**Evaluation of Death from Dengue Syndrome - Study of 60 Cases in a Tertiary Hospital of Bangladesh**	

Table S5 Quality Assessment Scores for Cross-Sectional Studies Based on the Newcastle–Ottawa Scale.

			**Selection**		**Comparability**	**Outcome**		
Author (Year)	Representativeness of the sample	Sample Size	Non-respondents	Ascertainment of the exposure (risk factors)	Comparability	Assessment of the outcome	Statistical test	Total score
Faruque (2012)	2	1	1	1	1	1	1	8/8
Amin (2022)	1	1	1	1	1	1	1	7/8
Muraduzzaman (2018)	2	1	1	1	1	1	1	8/8
Salje et al. (2019)	2	1	1	1	1	1	1	8/8
Hasan (2021)	2	1	1	1	1	1	1	8/8
Khan (2021)	1	1	1	1	1	1	1	7/8
Titir (2021)	2	1	1	1	1	1	1	8/8
Islam (2022)	1	1	1	1	1	1	1	7/8
Islam (2022)	1	1	1	1	1	1	1	7/8
Rafi (2022)	2	1	1	1	1	1	1	8/8
Yang (2023)	2	1	1	1	1	1	1	8/8
Yesmin (2023)	2	1	1	1	1	1	1	8/8

Rahim (2023)	1	1	1	1	1	1	1	7/8
Afroz (2024)	1	1	1	1	1	1	1	7/8
Chowdhury (2024)	1	1	1	1	1	1	1	7/8
**Hasan (2024)**	2	1	1	1	1	1	1	8/8
**Hossain (2024)**	1	1	1	1	1	1	1	7/8

Table S6 Quality Assessment of Retrospective Cohort Studies Using the Newcastle–Ottawa Scale

			Selection		Comparability	Outcome		
Author (Year)	Representativeness of the sample	Sample Size	Non-respondents	Ascertainment of the exposure (risk factors)	Comparability	Assessment of the outcome	Statistical test	Total score
Islam (2022)	2	1	1	1	1	1	1	8/8
Prattay (2022)	1	1	1	1	1	1	1	7/8
Hossain (2023)	2	1	1	1	1	1	1	8/8
Ahmad(2020)	1	1	1	1	1	1	1	7/8

Table S7 Quality Assessment of Case–Control Studies Using the Newcastle–Ottawa Scale

			Selection		Comparability	Outcome		
Author (Year)	Representativeness of the sample	Sample Size	Non-respondents	Ascertainment of the exposure (risk factors)	Comparability	Assessment of the outcome	Statistical test	Total score
Rahman (2022)	1	1	1	1	2	1	1	8/8

Supplementary Text S1 – Newcastle–Ottawa Scale criteria

Newcastle - Ottawa quality assessment scale for cohort studies (1)

Note: A study can be awarded a maximum of one point for each numbered item within the Selection and Outcome categories. A maximum of two stars can be given for Comparability
Selection
1) Representativeness of the exposed cohort
a) truly representative of the average _______________ (describe) in the community (1)
b) somewhat representative of the average ______________ in the community (1)
c) selected group of users e.g., nurses, volunteers
d) no description of the derivation of the cohort
2) Selection of the non-exposed cohort
a) drawn from the same community as the exposed cohort (1)
b) drawn from a different source
c) no description of the derivation of the non-exposed cohort
3) Ascertainment of exposure
a) secure record (eg surgical records) (1)
b) structured interview (1)
c) written self-report
d) no description
4) Demonstration that outcome of interest was not present at start of study
a) yes (1)
b) no
Comparability
1) Comparability of cohorts on the basis of the design or analysis
a) study controls for _____________ (select the most important factor) (1)
b) study controls for any additional factor (1) (This criterion could be modified to indicate specific control for a second important factor.)

Outcome
1) Assessment of outcome
a) independent blind assessment (1)
b) record linkage (1)
c) self-report
d) no description
2) Was follow-up long enough for outcomes to occur
a) yes (select an adequate follow up period for outcome of interest) (1)
b) no
3) Adequacy of follow up of cohorts
a) complete follow up - all subjects accounted for (1)
b) subjects lost to follow up unlikely to introduce bias - small number lost - > ____ % (select an adequate %) follow up, or description provided of those lost) (1)
c) follow up rate < ____% (select an adequate %) and no description of those lost
d) no statement

1. Newcastle - Ottawa quality assessment scale for case control studies [20]

Note: A study can be awarded a maximum of one point for each numbered item within the Selection and Exposure categories. A maximum of two points can be given for Comparability.

Selection

1) Is the case definition adequate?

a) yes, with independent validation (1)

b) yes, e.g., record linkage or based on self-reports

c) no description

2) Representativeness of the cases

a) consecutive or obviously representative series of cases (1)

b) potential for selection biases or not stated

3) Selection of Controls

a) community controls (1)

b) hospital controls0

c) no description

4) Definition of Controls

a) no history of disease (endpoint) (1)

b) no description of source

Comparability

1) Comparability of cases and controls on the basis of the design or analysis

a) study controls for _______________ (Select the most important factor.) (1)

b) study controls for any additional factor (1) (This criterion could be modified to indicate specific control for a second important factor.)

Exposure

1) Ascertainment of exposure

a) secure record (e.g., surgical records) (1)

b) structured interview where blind to case/control status (1)

c) interview not blinded to case/control status

d) written self-report or medical record only

e) no description

2) Same method of ascertainment for cases and controls

a) yes (1)

b) no

3) Non-Response rate

a) same rate for both groups (1)

b) non respondents described

c) rate different and no designation

Table S8. Demographic characteristics in the included studies

Author	**Year of publication**	Average Age (years)	Male (%)	Female (%)	Urban (%)	Rural (%)	Endemic/Outbreak

Islam et al.	2006	29.0	NA	NA	NA	NA	Outbreak
Faruque et al.	2012	19.8	NA	NA	51.0	49.0	Endemic
Dhar-Chowdhury et al.	2016	31.9	NA	NA	NA	NA	Endemic
Amin et al.	2022	31.0	60.6	39.4	87.0	13.0	Outbreak
Muraduzzaman et al.	2018	6.95	62.0	38.0	76.0	26.0	Endemic
Salje et al.	2019	NA	NA	NA	NA	NA	Endemic
Hasan et al.	2021	27.0	63.2	36.8	51.0	49.0	Outbreak
Hoque et al.	2021	NA	51.6	48.4	NA	NA	Endemic
Khan et al.	2021	8.8	55.0	45.1	NA	NA	Outbreak
Mahmood et al.	2021	26.15	60.5	39.5	100.0	NA	Outbreak
Titir et al.	2021	NA	61.5	38.5	NA	NA	Endemic
Islam et al	2022	34.0	55.2	55.2	NA	NA	Endemic
Islam et al.	2022	7.3	61.0	38.2	23.6	76.4	Endemic
Prattay et al.	**2022**	29.0	65.5	34.5	54.0	45.7	Outbreak
Rafi et al.	2020	33.0	70.0	30.0	46.0	54.0	Endemic
Rahman et al.	2022	NA	50.0	50.0	NA	NA	Endemic
Yang et al.	2023	NA	NA	NA	NA	NA	Outbreak
Yesmin et al.	2023	33.3	60.0	40.0	100.0	NA	Outbreak
Rahim et al.	2023	NA	NA	NA	100.0	NA	Endemic
Sami et al.	2023	29.0	66.6	33.4	79.9	20.1	Endemic
Afroz et al.	2024	34.5	48.3	51.7	89.2	10.8	Outbreak
Chowdhury et al.	2024	26.0	72.3	27.7	65.7	34.3	Outbreak
Hasan et al.	2024	NA	NA	NA	66.8	33.2	Outbreak
Hossain et al.	2024	33.0	79.5	20.5	100.0	NA	Outbreak

Table S9. Clinical profiles of dengue patients

Clinical characteristics	Amin et al. Total (n=297) n(%)	Faruque et al. Total (n=69) n(%)	Hasan et al. Total (n=553) n(%)	Islam et al. Total (n=100) n(%)	Islam et al. Total (n=478) n(%)	Islam et al. Total (n=123) n(%)	Khan et al. Total (n=190) n(%)	Mahmood et al. Total (n=542) n(%)	Prattay et al. Total (n=336) n(%)	Rafi et al. Total (n=319) n(%)	Yesmin et al. Total (n=369) n(%)	Rahim et al. Total (n=67) n(%)	Sami et al. Total (n=309) n(%)	Afroz et al. Total (n=176) n(%)	Chowdhury et al. Total (n=1978) n(%)
**Severe Symptoms**															
Dyspnea (Shortness of breath)	NA	NA	NA	NA	NA	11 (8.9)	31 (16.6)	NA	NA	NA	NA	NA	52 (16.9)	NA	NA
Dehydration	239 (82.1)	NA	31 (7.8)	NA	NA	NA	21 (11.9)	NA	6 (1.8)	NA	89 ()24.1)		NA	NA	NA
Gastrointestinal bleeding	NA	NA	NA	NA	9 (13.2)	NA	NA	NA	NA	NA	NA	10 (14.9)	NA	NA	NA
Gum bleeding	NA	NA	49 (10.1)	41	NA	NA	NA	11.1	3 (0.9)	25 (7.8)	NA	NA	NA	NA	NA
Persistent vomiting	NA	NA	NA	NA	NA	NA	NA	NA	NA	NA	61 (16.5)		114 (37.0)	48 (27.3)	NA
Ascites	NA	NA	NA	NA	NA	NA	NA	NA	NA	NA	33 (8.94)	9 (13.4)	NA	36 (20.1	69(3.5)
Melena (Blood in stool)	NA	NA	30 (5.4)	NA	NA	24 (64.9)	11 (5.8)	NA	NA	11.9	19 (5.2)		16 (5.2)	NA	NA
Body ache	79 (26.6)	16 (23.2)	NA	NA	NA	71 (57.7)	NA	NA	132 (39.3)	NA	NA	23 (34.3)	NA	NA	NA
Muscle pain	NA	2 (2.9)	NA	NA	NA	NA	NA	NA	NA	NA	NA	NA	NA	NA	NA
Joint pain (Arthralgia	87 (27.9)	5 (7.2)	25 (4.5)	91 (91.0)	NA	NA	75 (40.8)	NA	7 (2.1)	NA	NA	NA	80 (26.0)	NA	NA

Bone pain	NA	NA	NA	NA	260 (54.4)	NA		NA	NA	NA	NA	NA	NA	NA	
Retro-orbital pain	151 (50.8)	1 (1.4)	216 (39.1)		41 (8.6)	26 (21.1)	63 (34.4)	27 (5.0)	NA	150 (47.0)	132 (35.8))	NA	NA	35 (19.9)	156(7.9)
Back pain	218 (73.4)	NA	58 (10.5)	NA	NA	NA	66 (36.7)	NA	8 (2.4)	NA	104 (28.18)		NA	NA	NA
Neck pain	120 (40.4)	0	39 (7.1)		NA	NA	NA	NA	NA	NA	NA	NA	16 (5.2)	NA	NA
Sore throat	87 (29.3)		9 (1.6)		NA	NA	NA	NA	NA	NA	49 (13.3)			NA	NA
Arthralgia	NA	NA	NA	NA	NA	NA	NA	NA	NA	NA	20	NA	NA	NA	NA
Bleeding	NA	1 (1.4)	NA		NA	37 (30.1)			NA		NA	NA	NA	NA	NA
Hematuria (presence of blood in the urine)	NA	NA	3 (0.5)	NA	45 (09.4)	1 (2.7)	NA	NA	NA	NA	12 (3.3)		7 (2.3)	NA	NA
Gum bleeding	NA	NA	20 (3.6)	41 (41.0)	49 (10.3)	NA		36 (11.1)	NA	7.8	22 (5.96)		12 (3.9)	NA	132(6.7)
Epistaxis (Nasal bleeding)	NA	NA	9 (1.6)	2 (2.0)		3 (8.1)	12 (23.7)	NA	6 (1.8)	8 (2.5)	7 (1.9)		16 (5.2)	NA	NA
Gastrointestinal (GIT) bleeding	NA	NA	NA	50 (50.0)	13 (18.0)	NA		NA	NA	NA	NA	NA	NA	NA	NA
Hypotensive	17.6	NA	135 (25.0)	NA	NA	3 (2.4)		90	NA		NA	NA	NA	NA	NA
Edema and ascites	NA	NA	7 (1.3)	7 (7.0)	NA	5 (4.1)		NA	3 (0.9)		NA	NA	NA	NA	NA
Respiratory distress	46 (15.5)	NA	25 (4.5)	NA	NA	NA	NA	NA	5 (1.5)	7 (2.2)	NA	NA	NA	NA	NA

Pleural effusion (listed twice, needs verification)	NA	NA	10 (1.8)	NA	NA	3 (2.4)	NA	NA	NA	7 (2.19)	50 (13.5)	2 (29.9)	25 (14.2)	25(14.2)	96(4.8)
**Common Symptoms**														NA	NA
Fever	287 (96.6)	69 (100.0)	553(100.0)	100 (100.0)	452 (94.6)	123 (100.0)	190 (100.0)	505 (93.1)	301 (98.0)	295 (92.5)	369 (100.0)	67 (100.0)	308 (100.0)	153 (87.0)	NA
Headache	269 (90.6)	33 (47.8)	347 (62.7)	96 (96.0)	234 (49.0)	70 (56.9)	126 (67.7)	249 (45.9)	71 (21.0)	232 (72.3)	223 (60.4)	23 (23.9)	191 (62.0)	96 (54.6)	1330(67.2)
Chill	243 (81.8)	NA	NA	NA	NA	NA	NA	NA	NA	NA	NA	NA	222 (72.1)	NA	NA
Cough	118 (39.7)	NA	30 (5.4)	NA	119 (24.9)	NA	NA	NA	12 (3.6)	9 (2.8)		7 (10.4)	127 (41.0)	22 (36.7)	NA
Nausea/Weakness	NA	NA	NA	NA	NA	NA	141 (78.3)	NA	NA	NA	249 (67.5)	36 (53.7)	209 (67.9)	NA	844(43.0)
Skin rash	50 (16.8)	1 (1.0))	25 (4.5)	28 (28.0)	280 (58.6)	68 (53.3)	52 (27.7)	137 (25.3)	18 (5.4)	50 (15.7)	64 (17.3)	9 (13.4)	42 (13.6)	NA	326(16.5)
Body ache / Myalgia	26(59.0)	NA	NA	NA	324 (67.8)	NA		146 (26.9)	NA	228 (71.5)	73 (19.8)		NA	125 (71.0)	1519(76.8)
Jaundice	4 (2.7)	2 (2.9)	4 (0.7)	1 (1.0)	NA	NA	NA	NA	NA	NA	NA	NA	10 (3.2)	NA	NA
Anorexia	239 (80.5)	NA	385 (69.6)	93 (93.0)	32	NA		NA	60 (70.9)	NA	162 (43.9)	12 (17.9)	17.9	NA	NA
Abdominal pain	100 (33.7)	86.5	230 (41.6)	83 (83.0)	29.71	36 (29.3)	122 (64.9)	160 (29.5)	12	95 (30.0)	136 (36.9)	37 (55.2)	155 (50.3)	84(47.7)	284(14.4)
Diarrhea	115 (38.7)	NA	145 (26.2)	NA	57 (11.9)	24 (19.5)	81 (42.9)	107 (19.7)	26 (7.7)	131 (94.9)	134 (36.3)	6 (9.0)	79 (25.6)	56 (33.1)	514(26.0)
Decreased appetite	NA	NA	NA	NA	11.92	NA	151 (79.5)	19.7	NA	NA	36	9	26.6	NA	NA

Constipation	NA	NA	NA	NA	NA	NA	133 (72.7)	NA	NA	12 (3.76)	32 (8.67)		NA	NA	NA
Mouth sore for children	NA	NA	NA	NA	NA	NA	53 (28.3)	NA	NA	NA	NA	NA	NA	NA	NA
Nausea and Vomiting	227 (76.4)	NA	385 (69.6)	93 (93.0)	153 (32.01)	45 (36.6)	152 (80.4)	331(61.1)	99 (29.5)	138 (43.26)		36 (53.7)	196 (63.6)	48(27.3)	1068 (54.0)
